# Collectanea Medica: Consisting of Anecdotes, Facts, Extracts, Illustrations, &c.

**Published:** 1825-06

**Authors:** 


### COLLECTANEA MEDICA:

CONSISTING OF

ANECDOTES, FACTS, EXTRACTS, ILLUSTRATIONS, &c.

*Relating to the History or the Art of Medicine, and the Collateral Sciences.*

Floriferis, ut apes, in saltibus omnia libant,
Omnia nos, itidem, depascimur aurea dicta.

---

ART. I.—*On the Use of the Inclined Plane.* (From Mr. SHAW'S "*Further Observations on the Lateral or Serpentine Curvature of the Spine,*" *&c.*)

LYING on the inclined plane has of late years been considered so sure a method of curing all distortions, and also of preventing children from becoming crooked, that a plane is now considered a necessary appendage to the furniture of the nursery or school-room; and so much reliance has been placed on its good effects, that it is not unusual to hear a mother express surprise that her daughter should have become crooked, when she had been in the habit of lying every day for a certain time on the board.

The opinion generally entertained seems to be that, as the plane is used for the cure of girls whose spines are a little twisted, lying upon it must prevent those who are straight from becoming crooked. But this is a mistaken view, and there is little doubt that, from parents supposing that a child's figure will be preserved by lying for a certain time daily on the board, many circumstances of more importance are neglected.

The question may be put in this way. If a distortion could be remedied or prevented by a girl lying on her back for half an hour or an hour daily, would not lying in the same position for eight or ten hours during the night have the same, or even a better, effect? A moment's consideration must convince us that the effect of lying a short time on the board can be little, compared to that of lying in the same position during the whole night; and from this we may conclude that, if good is to be derived from lying in a certain manner, it is more important to regulate the mode in which a child lies in bed, than to insist that she should lie on the board, in the best position possible, for twenty minutes or half an hour twice or three times a-day. What is expected to be the consequence of a girl's lying for a short time on the plane? Is there any thing specific in the board, or is it more than the medium of affording rest? or is there any difference between the manner of lying on a board and that of lying on a mattress? Lying on a board is certainly more uncomfortable; and, perhaps, this is the reason why it is considered useful; for it seems to be an axiom, that if any thing connected with surgery is agreeable, it cannot be salutary.

The inclined plane, as it is commonly made, is good only so far as it gives rest and support to the body. Viewing it in this light, I see no reason why it should not be made comparatively comfortable, by cushions, that a girl may, by reclining on it, really be at rest, after a long and weary music-lesson or a fatiguing walk; for then is the time that the spine of a young person requires to be carefully managed.

It is generally supposed that the hard board is useful in assisting to press in the projecting shoulder; yet no opinion can be more incorrectly founded, or more likely to be the cause of injury to the patient; for it can be satisfactorily shown, by several cases, that lying on the hard board has increased the distortion. But, even setting aside the question of its being hurtful, and the absurd prejudice that because a board is uncomfortable it must be useful, and that if agreeable it must be injurious, we may assume that it is unnatural for any person, and more particularly a delicate girl, to lie on a hard board. Even animals make some sort of bed to rest upon.

I will not here enter into a description of the manner in which a girl who is a little distorted, may be constrained to lie in a certain position while in bed, (which is of more importance than the question how she lies during the time she may be on the board,) as it is given in the chapter, in the preceding volume, " on the Mode of treating the confirmed Lateral Curvature;" I shall at present only say, that a girl, who has the slightest tendency to become distorted, should sleep on a firm mattress, and with little or no pillow. The bad effects of a child habitually lying on a feather-bed, and with a high pillow, may be understood by the sketch given in illustration of one of the causes of distortion.

The practice of laying a patient on the back, and in an inclined or horizontal position, for months, and even for years, which has of late prevailed as a method of cure for all kinds of curvature of the spine, and more particularly the lateral, has been founded on the idea that the distortion depends on an undue contraction of certain muscles of the spine, and on a diseased state of the vertebræ. Taking this view of the cause of distortion, it was imagined that, by keeping a patient constantly at rest on an inclined or horizontal plane, the irritation proceeding from the supposed disease of the bones would be relieved; and the muscles of both sides being kept in a state of quietude, would be gradually reduced to the same standard of strength, so that in a certain time the equilibrium in their actions would be restored. But the ideas on which this mode of treatment has been founded are completely erroneous; and there are numerous facts to prove that, when put into practice, it has completely failed. Is it not surprising that keeping the body in one position should have been proposed as the best method of curing a defect in the spine, after the admission that the distortion is frequently consequent on weakness, and that if any part of the body be allowed to lie unexercised, it becomes deteriorated? for this plan is, of all others, the most effectual in rendering the body weak, and in preventing those muscles on which the support of the spine depends from performing their natural functions. The bad effects of such a method of treatment are gradually becoming evident, and the use of the inclined plane is quickly falling into disrepute; for, even where it at first seems to be useful, (as in cases of slight distortion attended with great debility), it is found that, although the girl may, perhaps, become straighter after having been confined to the horizontal position for months, she does not, after a time, gain strength, but, on the contrary, becomes so weak as to be scarcely able to walk or stand; and, when she attempts to sit up without some artificial support, she sinks almost double, or, at least, into a state worse than she was in when she first lay down.

So many instances of the complete failure of this mode of treatment in lateral curvature have come under my own observation, that the mere enumeration of them might be considered by many sufficient to prove the inefficacy of the system; but I suspect that even the detail of the cases would not convince others, as I know, from experience, that it is as difficult to alter the opinions of those who have taken a different view of this question, as to remove the prejudices of parents. I have little doubt that the good effects of the inclined plane in *other* diseases of the spine, will be brought forward to show that the system has been successful. However, as the question is not whether it has been useful in *some* affections of the spine, but whether it has been useful in cases of *common lateral* or *serpentine curvature*, I shall give a few examples of the consequences that I have found to proceed from it. These examples, and the arguments that may be deduced from an observation of the natural and diseased functions of the parts connected with the spine, will perhaps be considered, by those who are unprejudiced, as sufficient proof that the plan will invariably fail in cases of common lateral curvature.

In this discussion, I shall be obliged to question the accuracy of opinions which have been given in cases of distortion by several eminent practitioners; but I trust that the cases I shall now bring forward, and the numerous and indisputable facts that are offered in this and the preceding volumes, to prove that there is a much greater variety in the affections of the spine than was supposed to exist, (or, at least, than had been described,) will save me from the imputation of presumption.

I shall now select only four instances to exemplify the failure of the system of treatment by the inclined plane in lateral curvature, and the bad effects that have resulted from the supposition that the principles on which the practice was pursued are correctly founded. It may, perhaps, not be irrelevant to mention, that the rank in society of each individual whose case is stated, was such as to render it improbable that they should have continued long and seriously ill without having the best advice the country afforded; from which it is fair to conclude that the system they were put upon had met with the approbation of men who stand high in the profession.

The first instance I offer was the daughter of a gentleman, who was in the habit of spending the winter in town with his family, and who had consequently the opportunity (of which I know he availed himself) of having advice from the leading men in the profession.

I found the young lady, who was about fifteen years of age, reduced to such a state of weakness, that, when she attempted to rise, that I might examine her spine, she fainted: she was then unable to walk, or even to stand without support. As far as I could judge from her history, this debility was owing solely to long confinement in one position: she had lain on her back for two years. In this case, the system of restraint, and of preventing all action of the muscles of the back, had been carried to a degree scarcely credible; for, when she went to bed, or from that to her couch, she had always either been carried or rolled off from the one to the other. Instead of the curvature of the spine having been removed by this system of treatment, it had been increased: indeed, it was scarcely possible for any one to be in a worse condition. The ribs were distorted to an extraordinary degree; and this, I have no doubt, was caused by the constant pressure of the shoulder against the board, which I have, in the preceding volumes, shown to be as hurtful as the pressure made by machinery. It appeared that her debility was the effect of confinement, not of disease; for, having put her on an entirely different plan of treatment, she became, *in the course of a few weeks*, as robust and active as any girl of her age.

The second instance was the daughter of a gentleman in a neighbouring county. From the history given by her mother, I learned that she had at first merely the slight lateral curvature, which should not have confined her to her couch for a day. When I saw her, she had lain on her back on the plane for eighteen months: she could, with some difficulty, walk across the room, but could not stand without being supported. The spine appeared only a little distorted while she lay on her face, but it became completely curved when she stood up, and the ribs were more compressed than we find them in cases where this practice has not been pursued; so that I have no hesitation in asserting, that the distortion in this instance was also much increased by the confinement to the plane.

This young lady had all the appearance of full health and of being strong; but the appearance was deceptive, for it was produced by fat, not by healthy and vigorous muscle, as might be proved by taking hold of her arms, and still more by the tottering manner in which she moved when raised from her couch.

That this debility was, as in the last case, the effect of the confinement, and not of any disease, may be inferred from the following circumstance:—

After a consultation on the state of her spine, her father went into another room, to hear the opinions of the surgeons. On coming back, he found his daughter skipping about the room, dancing the steps of a quadrille. "O papa," says she, "I know I am to be laid on my back for two years, so I am taking my last quadrille!" This shows that there was not at first any disease, nor even any tenderness of the vertebræ, which should have required rest; and yet this fine girl was not only condemned to lie on the plane for a year and a half, but her spine was even repeatedly blistered, to remove a supposed disease of the vertebræ. I need not enter into a description of the plan of treatment which was followed by this young lady, as it was nearly the same as that described in the chapter on the mode of treating confirmed lateral curvature. By pursuing the system steadily, she quickly acquired strength; and her figure was in a few months so much improved, that her father, on coming to town to see her, could scarcely discover any distortion.

The subject of the third case was a gentleman, who was more than twenty years of age, and had been suffering from distortion for nearly twelve years. From the advice he had received, he had so completely confined himself to his couch, that he had scarcely left it five minutes, at any one time, during several months preceding my visit, (he even slept on it,) and for several years he had never risen from it, unless he was at the same time supported by one of the collars that are used by Mr. Chesher. When I first saw him, he could not raise himself to receive me; and, on begging him to show me how long he could support himself in the sitting posture, he made the attempt, but could not continue in it above half a minute, without suffering from a dreadful sense of suffocation. While he lay on his face, the lumbar part of his spine appeared straight, but it became twisted the moment he attempted to sit up. I immediately changed the plan of treatment; during the course of which some very curious phenomena, with regard to the action of certain muscles, occurred: these I have endeavoured to explain in the chapter on the means of remedying stooping. The system pursued was so successful in restoring strength, that, in the course of a few months, this gentleman, who had been confined to his couch, or encased in iron instruments, for nearly half his life, was enabled to rise, to throw aside all his artificial supports, and to partake of the amusements both of town and country.

The fourth instance is particularly interesting and important, as illustrating the danger of erroneous theories, when they are supported by the authority of eminent men. The patient was the daughter of a country banker, and nearly related to some of the highest medical authorities in England. From the history given to me by her mother, I concluded that her spine had been at first affected nearly in the same manner as those of some delicate children, and which, by proper management, may almost always be remedied. But, unfortunately for this young lady, as soon as it was discovered that her spine was distorted she was laid on her back; and, notwithstanding a confinement to the plane for years,—or, I should rather say, in consequence of this confinement,—the curvature of the spine became gradually worse, and, when I saw it, was almost irremediable.

Although her parents had observed an obvious difference for the worse, when the spine was examined from time to time, they were induced to persevere, on finding that the plan was approved of by some of the most eminent men in the profession, and on being flattered by repeated assurances held out to them (as I have known held out to others,) that their daughter would get up in a few months quite recovered. But, often as this promise has been made, I believe it was never yet fulfilled in a case of common lateral curvature; and in support of this assertion I have the authority of one, whose opportunities of seeing such cases have certainly been greater than those of any other man. I allude to Sir Astley Cooper, who told me, while in consultation on the case of this young lady, that he did not know a single instance of a girl being cured by that mode of treatment.

There is a common opinion, but a very erroneous one, and for which there is no foundation in fact, that the spine becomes fixed when a girl arrives at a certain age. It is this deceptive hope which has induced many a poor girl to confine herself to the plane for years. Every one must have met with instances where the distortion of the spine has increased after the age of twenty-one.

The above plan of treatment is to be reprobated in cases of lateral curvature, as it tends to increase rather than diminish the curvature and the original causes of distortion. But it is also objectionable in other respects, which are more important than the condition of the figure. It almost invariably injures the health. Girls who have been long confined to the reclining board are delicate, and liable to all the worst symptoms of hysteria. Indeed, I have known a young lady have so many of the symptoms of disease of the heart, that she was treated accordingly; although there was little doubt, from the history of their accession, and of the manner in which they were relieved, that they altogether proceeded from the weakness caused by the long confinement to one position. It is scarcely necessary to make any remarks on the danger of the internal organs suffering from the neglect or unwillingness of the patient to attend to the regular performance of their natural functions. An instance of a most serious organic disease in one of the pelvic viscera from this cause, is given on the best authority.

In opposition to this reasoning, the outline of which was given in the preceding volume, it has been stated that girls who have been long confined to the plane look fat and plump. They certainly sometimes appear so; but they are, in fact, in a relaxed state, and their muscles, when examined, are found to be soft and doughy, without any of that vigorous tone which always accompanies good health. We need seek no better proof of this than the inability of patients in this condition to keep the spine in an erect position, or to walk without support. Girls are often in such a state of weakness, from the same causes which produce the distortion, that they may be relieved by the recumbent position. But if this mode of relief be continued for any time, even in those cases where it has removed the pain in the chest and back, all the bad effects which have been already described will take place. The cases in which the health and strength are improved by lying on the inclined plane for weeks or months, are quite different from those at present under consideration.

I now proceed to make some observations on a subject, which is, in my opinion, of the first importance in considering the mode of practice to be followed in affections of the spine:—Does the pain which is usually felt in some parts of the spine, in cases of lateral curvature, depend on a disease of the bones, or inflammation of the ligaments? or is it merely the consequence of certain causes which may speedily be removed? Since writing the chapter in the preceding volume, "On the Inquiry into the Opinion that Lateral Distortion is caused by Disease of the Vertebræ," I have seen several patients who had suffered severely from the nature and cause of the pain having been mistaken. A young lady was confined to the horizontal position nearly a year, and repeatedly blistered, because a weary and dull pain in the lumbar part of the spine had been considered symptomatic of a commencing disease of the bones. How long she might have been kept in that position, is scarcely possible to imagine; for as, at the end of four months, she felt the same pain when she attempted to walk, it was supposed that the inflammation was not subdued, and she was accordingly ordered to submit six months longer: and, even at the expiration of that time, matters being still in the same state, she was laid a third time on the board. Soon after this I saw her, and having learned the history of her case, and made a careful examination of her spine, I was so much satisfied of there never having been any disease of the vertebræ, that, although the same pain was felt when she sat up, I put her on a plan of treatment very different from what had been pursued. In the course of a short time the pain ceased, and she became rapidly strong, and improved in appearance. But, notwithstanding the issue of this case, it would be a more serious error to mistake the pain consequent on inflammation of the bones, for that which is usually attendant on lateral distortion. When the vertebræ are actually diseased, there is generally a train of symptoms, in addition to the pain, which will assist us in discovering the nature of the case. It is, however, sometimes difficult to determine whether the pain is merely the effect of weakness or of a slight inflammation; and this difficulty I have found principally to present itself in cases of distortion in boys: for, as they are so little exposed to the causes which produce distortion in girls, we are led to suspect that those who become crooked have a peculiar weakness in their osseous system, and therefore that their vertebræ may be more than usually liable to the scrofulous inflammation.

But painful sensations in the back may depend on a variety of causes. The pain may arise from pressure on some of the viscera. An instance of this is related at page 200 in the preceding volume, but in that case the distortion was to a great degree. When the distortion is slight, it may depend on someof the nerves being compressed in their passage from the spinal canal. I have little doubt that this was the cause of an almost indescribable pain which a young gentleman, who was for some time under my care, felt when he sat in a particular position; for the pain always ceased while his shoulders were supported, and gradually left him as the spine became straight.

There is one cause of these painful sensations, to which we should particularly attend. A young lady from the neighbourhood of Dublin had a displacement, or rather a malformation, of the spinous processes of two of the vertebræ, such as I have described at page 90 in the preceding volume. When one of these vertebræ was pressed upon in a particular manner, a pain darted through her shoulder. As she was of rather a delicate constitution, and as some of her relations had been great sufferers from disease of the spine, her mother became alarmed, and brought her to London. The cause of the apparent distortion was easily explained, but I could not discover the source of the pain. However, it happened that about the same time I was consulted on the case of a young lady, who suffered nearly in a similar manner, when a particular part of the spine was touched. On comparing the symptoms in the two cases, I was led to suspect that the pain was occasioned by a branch of a nerve being pressed against one of the processes of the vertebræ. I examined the course of the nerves at this part more carefully than I had previously done, and found branches of the dorsal nerves passing very near the same processes as those which, when pressed upon, had given the sensation of pain: it is, therefore, probable that the process may occasionally pass under the nerve, and thus subject the nerve to pressure. But, even although the nerve be not entangled with the process, the same pain may be felt during an examination of the back; for, as the nerves (and particularly that between the fifth and sixth dorsal vertebræ) lie between the spinous processes and the angles of the ribs, they will be thrown so much forward on the convexity of the curve when the spine is distorted, as to be directly exposed to the touch. Indeed, I now find that, by pressing in a particular manner, a pain, similar to that described above, may be produced in almost every case of lateral curvature of the spine.

The nature of the pain caused in this way is so similar to that described by some authors, as occasioned by disease of the vertebræ, that it may have sometimes been considered an unequivocal symptom of disease of the vertebræ, and the patient treated accordingly. We cannot help suspecting that this has happened, when we reflect on the many instances where patients have been confined to the horizontal position, and blisters or issues have been applied on each side of the spine, merely because there has been a little irregularity in the shape of the column, and a certain degree of pain felt upon pressure.

The examples given in the Appendix to the preceding volume, make it unnecessary to show that I attach due importance to a tenderness in the course of the spine, when it is combined with certain other symptoms.

It is scarcely requisite, I trust, for me to state that, in my strictures on the inclined plane, I have not intended more than to object, first, to its being considered as the only thing necessary towards the cure of distortion; and, secondly, to the use of it, without remission, for months. In the chapters in the preceding volume that are descriptive of what I believe to be the best means of treating distortions, it will be found that the use of the inclined plane is considered essential to the cure of lateral curvature. And there cannot be a doubt that a young and delicate girl may be much the better of occasionally lying down: when she is growing quickly, and is at the same time delicate, the weight of the upper part of the body is obviously so much more than the lumbar vertebræ can support beyond a certain time, that common sense dictates the necessity of giving occasional ease and rest to the muscles of the spine; and this cannot be more effectually or more easily done than by lying down, either on the inclined plane or on a couch, where she may have room, while in the horizontal position, to shift her body as she pleases.

It may, in conclusion, be affirmed that, beneficial as the use of the inclined plane is in many instances, it has, like other valuable remedies, been very improperly employed, from erroneous notions having been entertained of its effects, and more especially from practitioners not having been aware of the great variety of affections to which the spine is liable.

